# Supplementary material for: Design, synthesis and antimicrobial activities of novel 1,3,5-thiadiazine-2-thione derivatives containing a 1,3,4-thiadiazole group
Source: PeerJ. 2019 Sep 6;7:e7581. doi: 10.7717/peerj.7581 (PMC6733239; doi:10.7717/peerj.7581)
Supplement: Supplemental Information 1 [file peerj-07-7581-s001.docx]

**Supplemental Information**

**Design, synthesis and antimicrobial activities of novel** **1,3,5-thiadiazine-2-thione derivatives containing a 1,3,4-thiadiazole group**

**Jinghua Yan^1^, Weijie Si^1,2^, Haoran Hu^1^, Xu Zhao^1^, Min Chen^1*^ and Xiaobin Wang^1^**

^1^ Jiangsu Key Laboratory of Pesticide Science, College of Sciences, Nanjing Agricultural University, Nanjing 210095, China

^2^ Key Laboratory of Monitoring and Management of Crop Diseases and Pest Insects, Ministry of Agriculture, Nanjing Agricultural University, Nanjing 210095, China

Corresponding Author: Min Chen (chenmin@njau.edu.cn).

**Table of Contents**

[1. Physical properties and spectral data of compounds **3a**–**3d** 1](#_Toc13564953)

[2. Physical properties and spectral data of compounds **4a**–**4d** 1](#_Toc13564954)

[3. ^1^H NMR, ^13^C NMR and HRMS spectra of title compounds **5**–**8** 2](#_Toc13564955)

[4. Crystal data of compound **8d** 26](#_Toc13564956)

[5. The *in vitro* antimicrobial activity of the title compounds **5**–**8**. 27](#_Toc13564957)

[6. References 29](#_Toc13564958)

# 1. Physical properties and spectral data of compounds 3a–3d


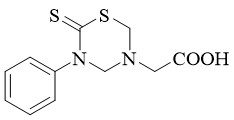


2-(5-Phenyl-6-thioxo-1,3,5-thiadiazinan-3-yl)acetic acid (**3a**) *(Wang et al., 2019)*: white solid, m.p.143–144^o^C, yield 48%; ^1^H NMR (400 MHz, DMSO-*d*_6_) *δ* 12.81 (s, 1H, COOH), 7.46 (t, *J* = 7.7 Hz, 2H, PhH), 7.35 (t, *J* = 7.4 Hz, 1H, PhH), 7.25–7.18 (m, 2H, PhH), 4.75 (s, 2H, thiadiazine-H), 4.69 (s, 2H, thiadiazine-H), 3.78 (s, 2H, CH_2_).


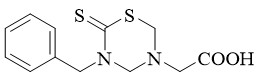


2-(5-Benzyl-6-thioxo-1,3,5-thiadiazinan-3-yl)acetic acid (**3b**): white solid, m.p.144–145^o^C, yield 71%; ^1^H NMR (400 MHz, DMSO-*d*_6_) *δ* 12.60 (s, 1H, COOH), 7.37 (s, 2H, PhH), 7.36 (s, 2H, PhH), 7.31 (dt, *J* = 13.2, 4.3 Hz, 1H, PhH), 5.28 (s, 2H, CH_2_), 4.57 (s, 2H, thiadiazine-H), 4.49 (s, 2H, thiadiazine-H), 3.38 (s, 2H, CH_2_).


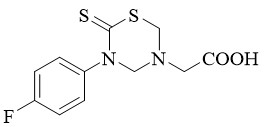


2-(5-(4-Fluorophenyl)-6-thioxo-1,3,5-thiadiazinan-3-yl)acetic acid (**3c**): white solid, m.p.137–138^o^C, yield 46%; ^1^H NMR (400 MHz, DMSO-*d*_6_) *δ* 12.79 (s, 1H, COOH), 7.33–7.24 (m, 4H, PhH), 4.75 (s, 2H, thiadiazine-H), 4.68 (s, 2H, thiadiazine-H), 3.78 (s, 2H, CH_2_).


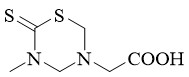


2-(5-Methyl-6-thioxo-1,3,5-thiadiazinan-3-yl)acetic acid (**3d**) (*Wang et al., 2018*): white solid; m.p.129–130°C; yield 67%; ^1^H NMR (400 MHz, DMSO-*d*_6_) *δ* 12.71 (s, 1H, COOH), 4.54 (s, 2H, thiadiazine-H), 4.52 (s, 2H, thiadiazine-H), 3.57 (s, 2H, CH_2_), 3.36 (d, *J* = 6.5 Hz, 3H, CH_3_).

# 2. Physical properties and spectral data of compounds 4a–4d


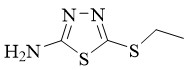


5-(ethylthio)-1,3,4-thiadiazol-2-amine(**4a**) (*Gao et al., 2013*) : yellow solid, m.p.214–216°C, yield 80%; ^1^H NMR (400 MHz, DMSO-*d*_6_) *δ* 7.28 (s, 2H, NH_2_), 3.05 (q, *J* = 7.3 Hz, 2H, CH_2_), 1.28 (t, *J* = 7.3 Hz, 3H, CH_3_).


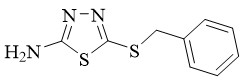


5-(benzylthio)-1,3,4-thiadiazol-2-amine (**4b**): yellow solid, m.p.161–163°C, yield 79%; ^1^H NMR (400 MHz, DMSO-*d*_6_) *δ* 7.34 (t, *J* = 7.3 Hz, 5H, PhH), 7.28 (t, *J* = 7.6 Hz, 2H, NH_2_), 4.30 (s, 2H, CH_2_).


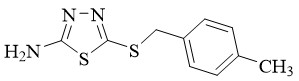


5-((4-methylbenzyl)thio)-1,3,4-thiadiazol-2-amine (**4c**): yellow solid, m.p.175–177°C, yield 81%; ^1^H NMR (400 MHz, DMSO-*d*_6_) *δ* 7.28 (s, 2H, NH_2_), 7.23 (d, *J* = 8.0 Hz, 2H, PhH), 7.13 (d, *J* = 7.9 Hz, 2H, PhH), 4.25 (s, 2H, CH_2_), 2.28 (s, 3H, CH_3_).


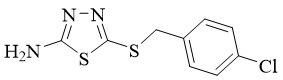


5-((4-chlorobenzyl)thio)-1,3,4-thiadiazol-2-amine (**4d**): yellow solid, m.p.163–165°C, yield 77%; ^1^H NMR (400 MHz, DMSO-*d*_6_) *δ* 7.41–7.34 (m, 4H, PhH), 7.30 (s, 2H, NH_2_), 4.29 (s, 2H, CH_2_).

# 3. ^1^H NMR, ^13^C NMR and HRMS spectra of title compounds 5–8


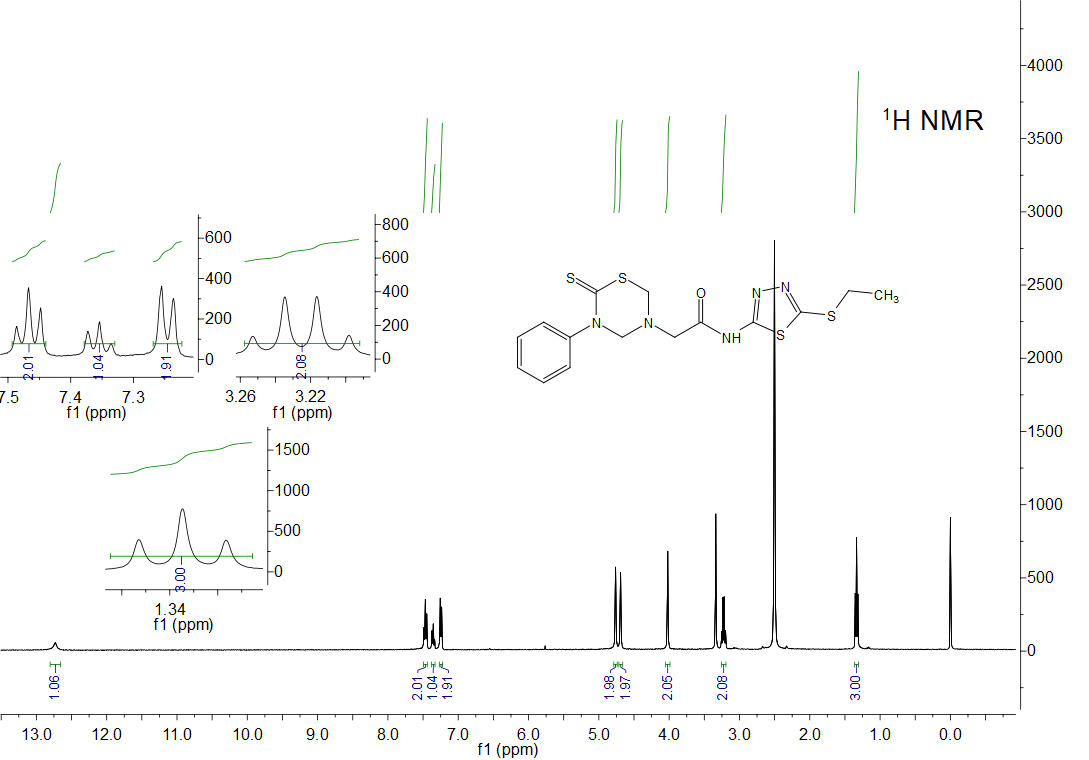


*Fig. S1* ^1^H NMR spectrum of title compound **5a**


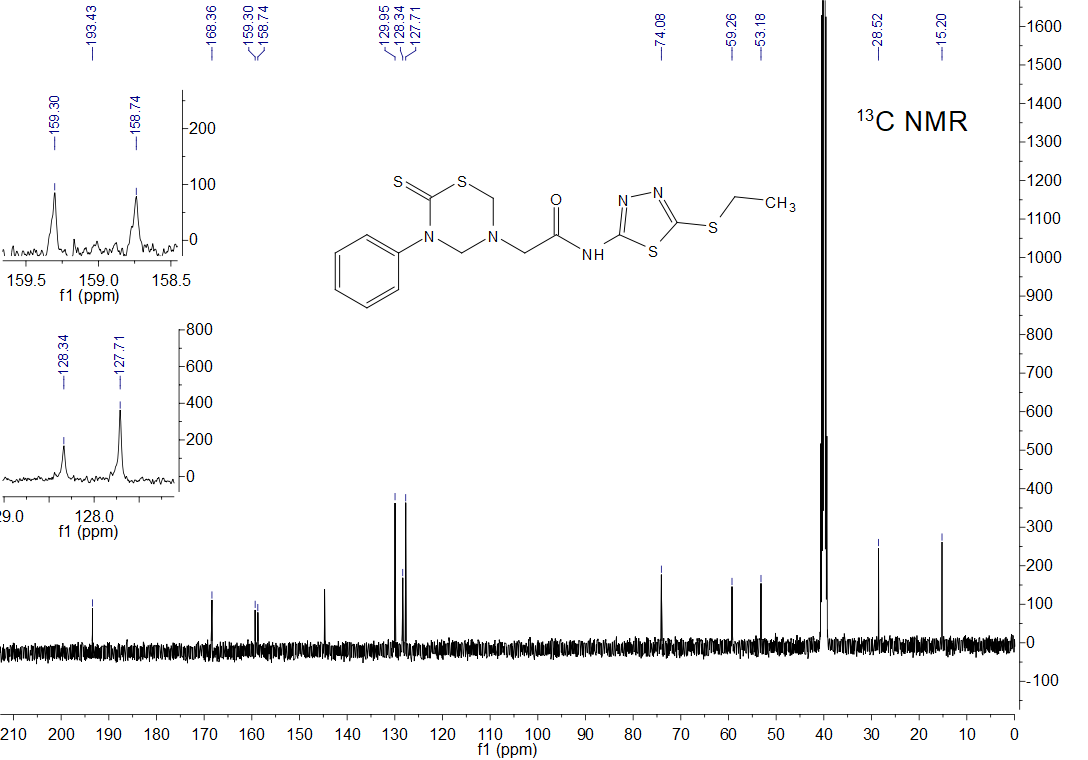


*Fig. S2* ^13^C NMR spectrum of title compound **5a**

*Fig. S3* HRMS spectrum of title compound **5a**


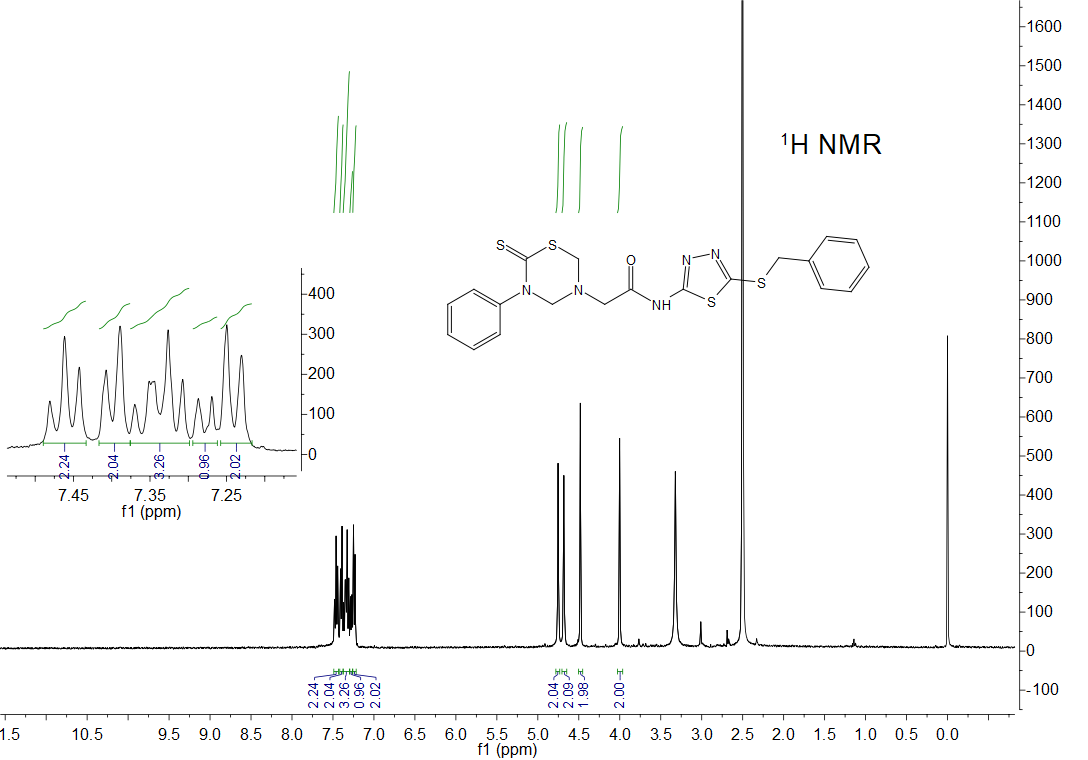


*Fig. S4* ^1^H NMR spectrum of title compound **5b**


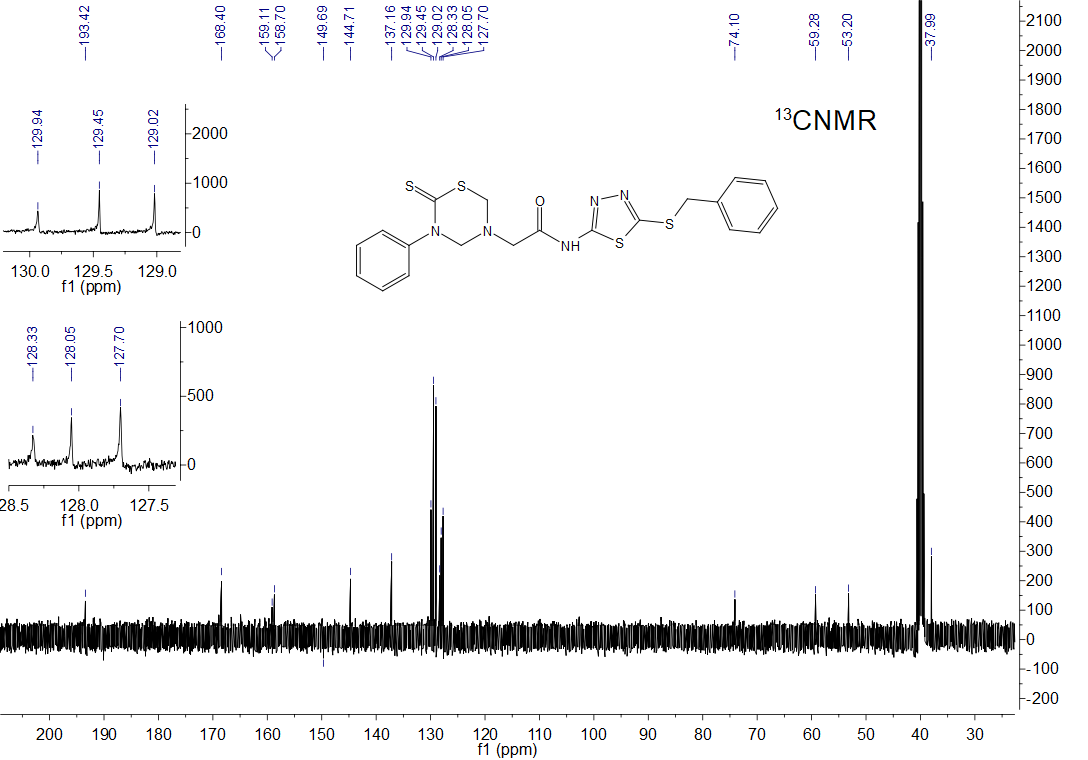


*Fig. S5* ^13^C NMR spectrum of title compound **5b**

*Fig. S6* HRMS spectrum of title compound **5b**


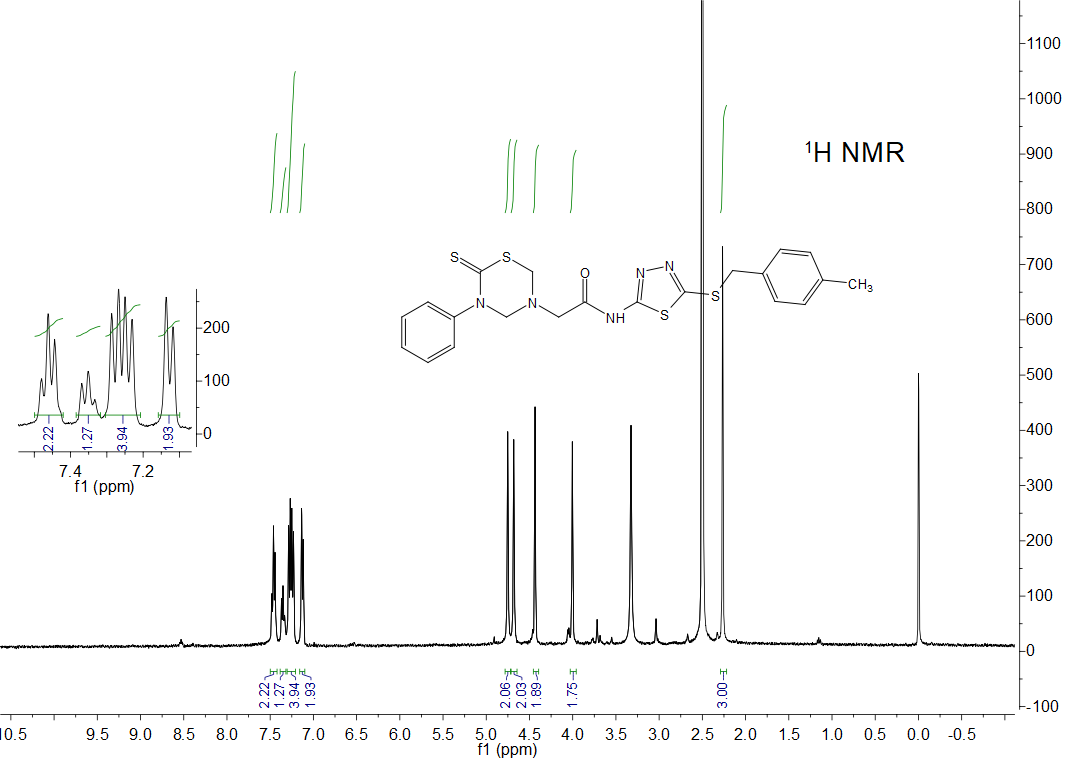


*Fig. S7* ^1^H NMR spectrum of title compound **5c**


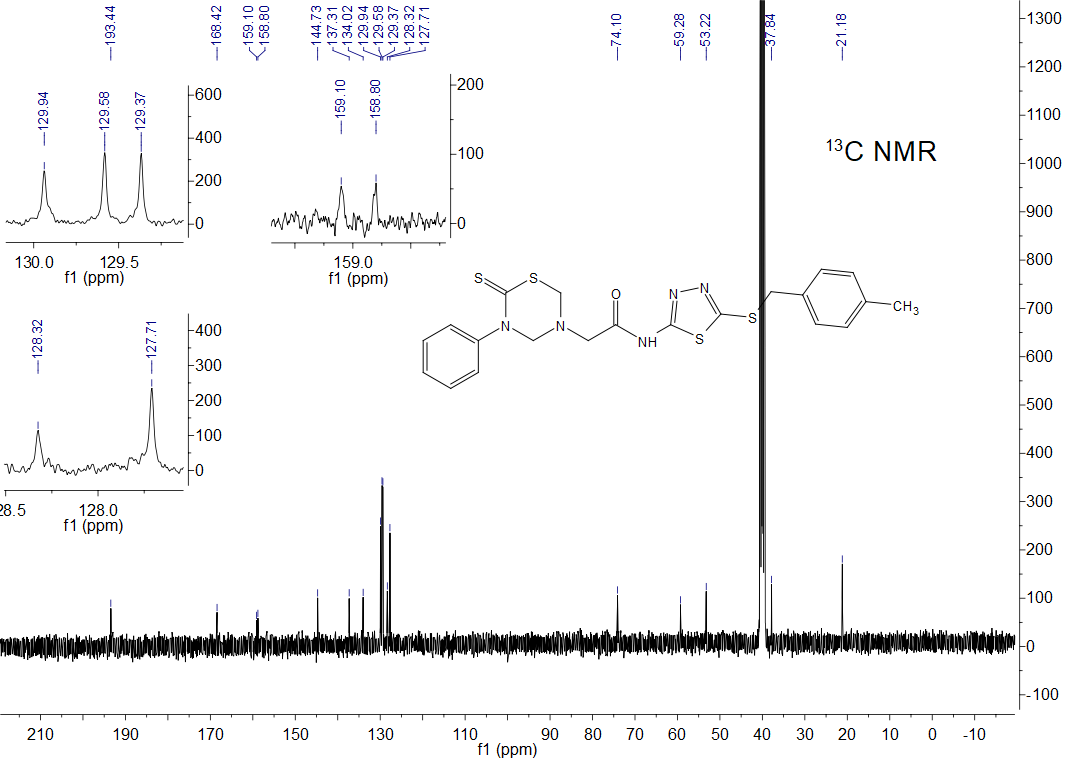


*Fig. S8* ^13^C NMR spectrum of title compound **5c**

*Fig. S9* HRMS spectrum of title compound **5c**


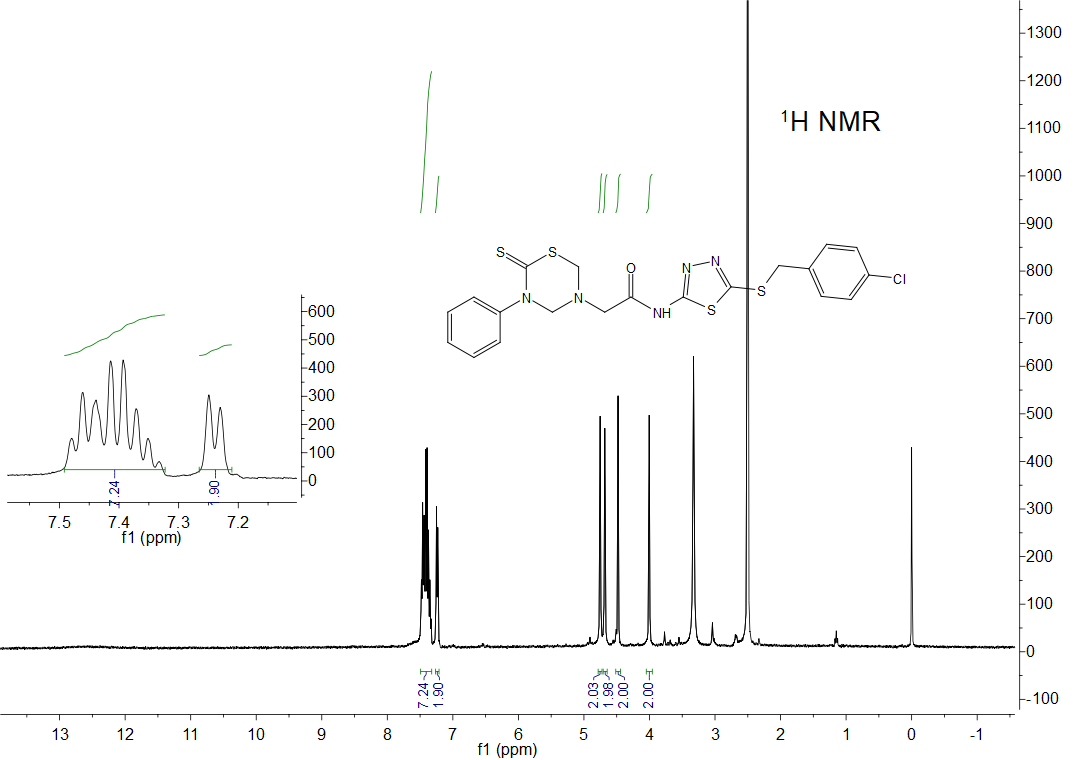


*Fig. S10* ^1^H NMR spectrum of title compound **5d**


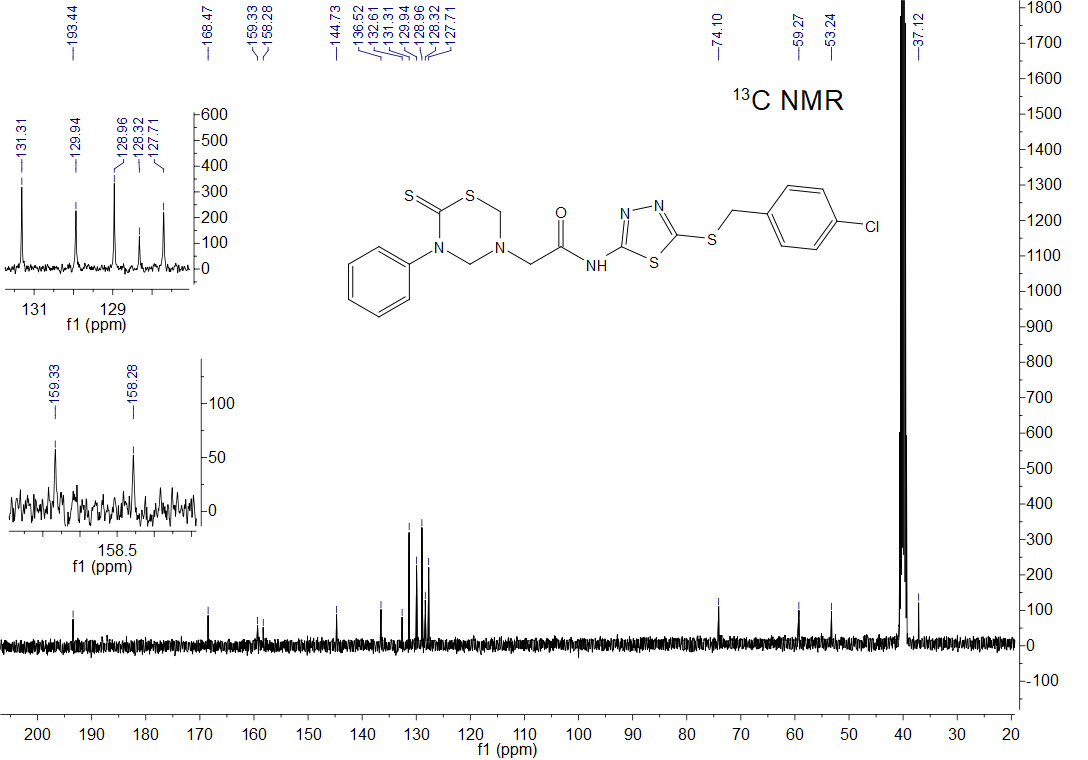


*Fig. S11* ^13^C NMR spectrum of title compound **5d**

*Fig. S12* HRMS spectrum of title compound **5d**


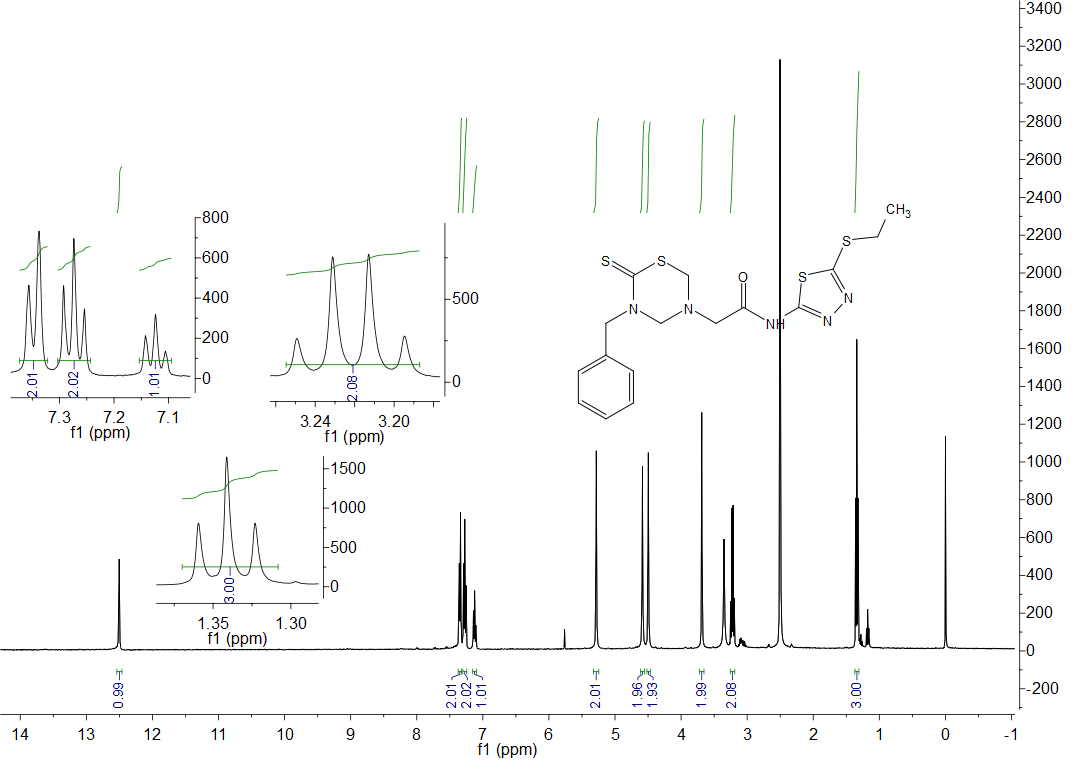


*Fig. S13* ^1^H NMR spectrum of title compound **6a**


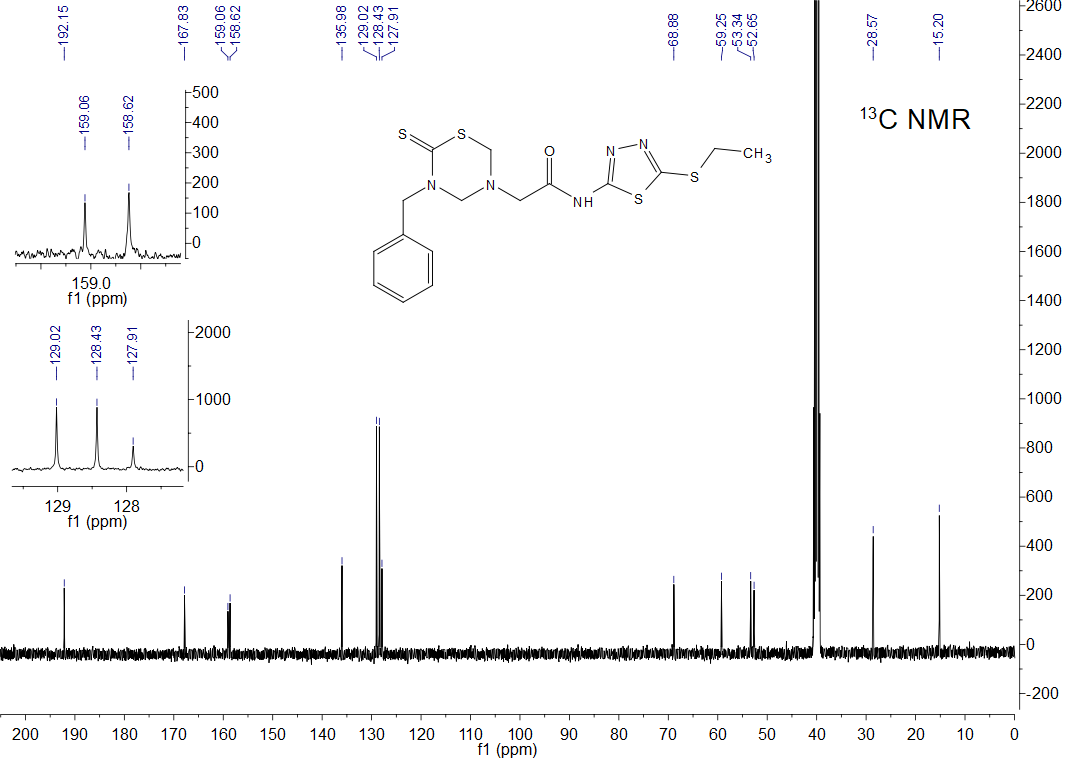


*Fig. S14* ^13^C NMR spectrum of title compound **6a**

*Fig. S15* HRMS spectrum of title compound **6a**


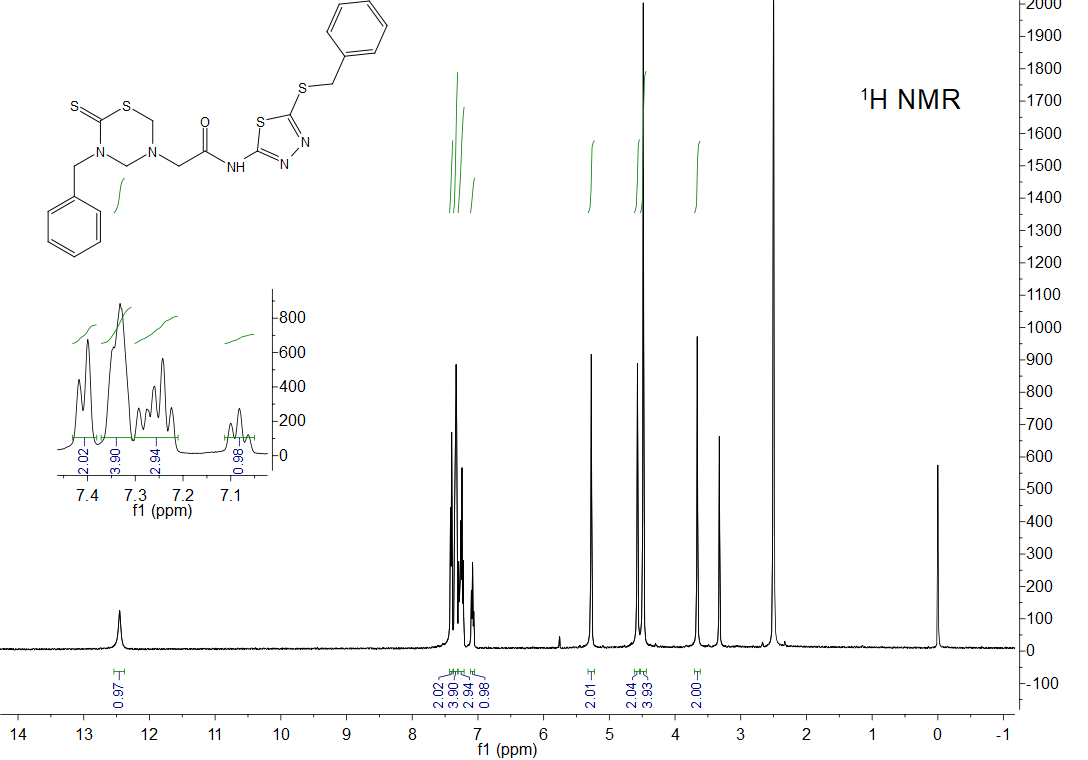


*Fig. S16* ^1^H NMR spectrum of title compound **6b**


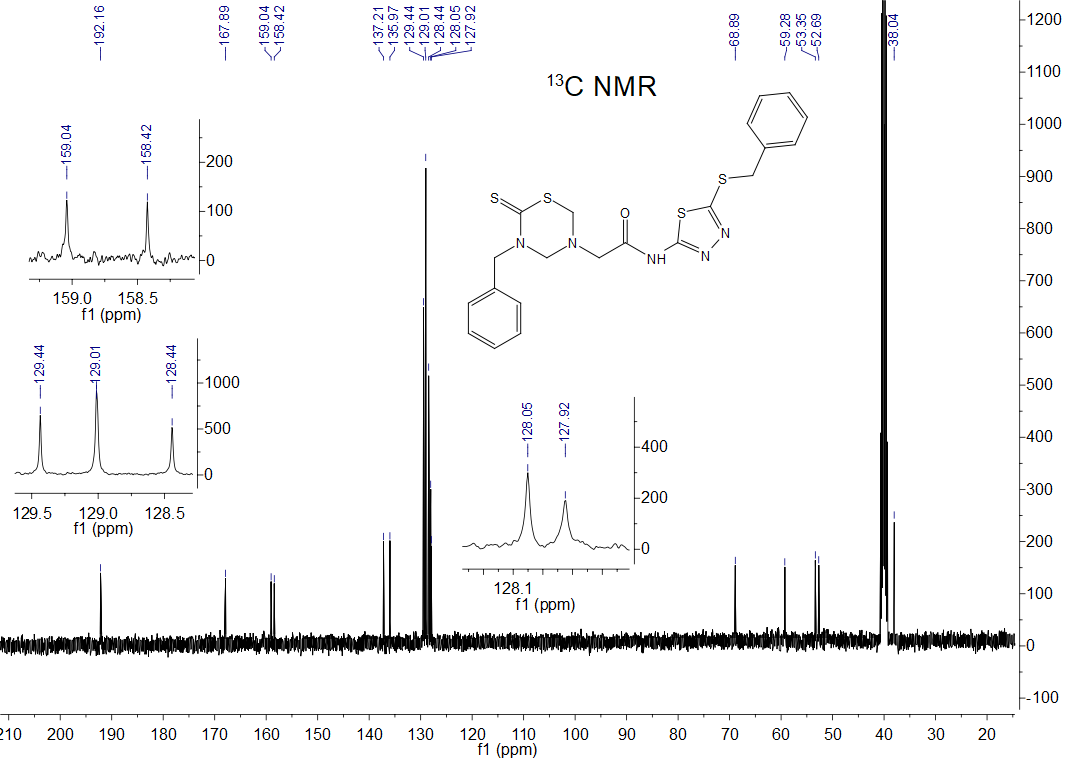


*Fig. S17* ^13^C NMR spectrum of title compound **6b**

*Fig. S18* HRMS spectrum of title compound **6b**


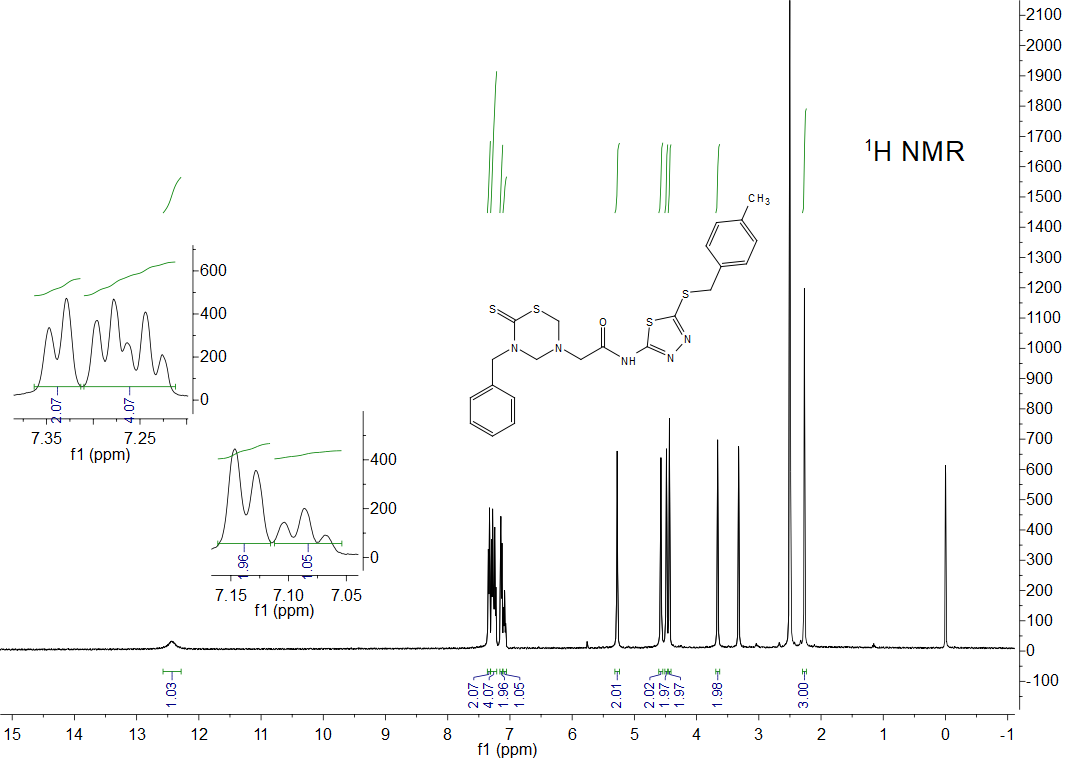


*Fig. S19* ^1^H NMR spectrum of title compound **6c**


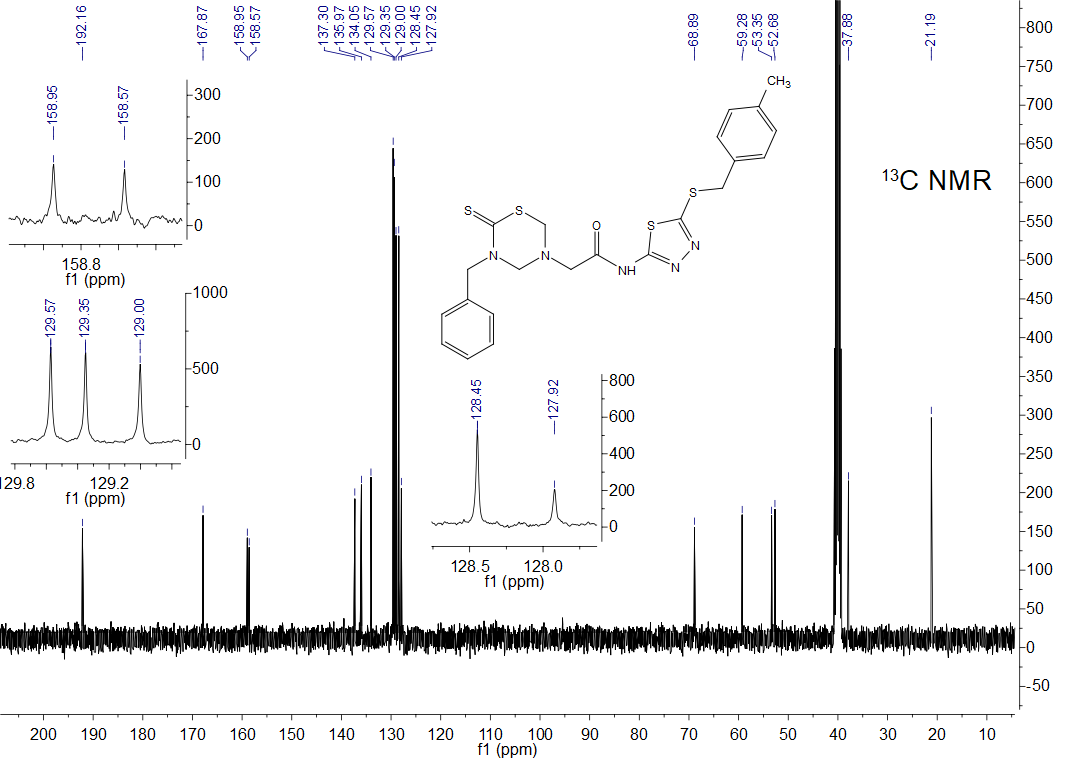


*Fig. S20* ^13^C NMR spectrum of title compound **6c**

*Fig. S21* HRMS spectrum of title compound **6c**


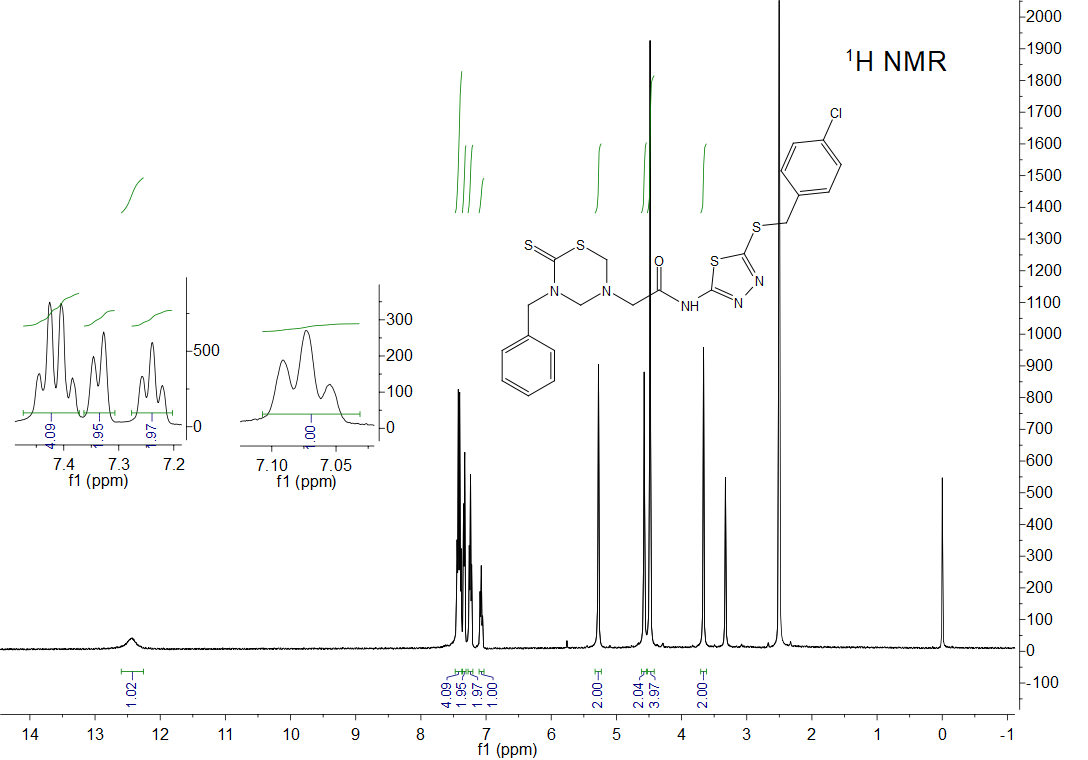


*Fig. S22* ^1^H NMR spectrum of title compound **6d**


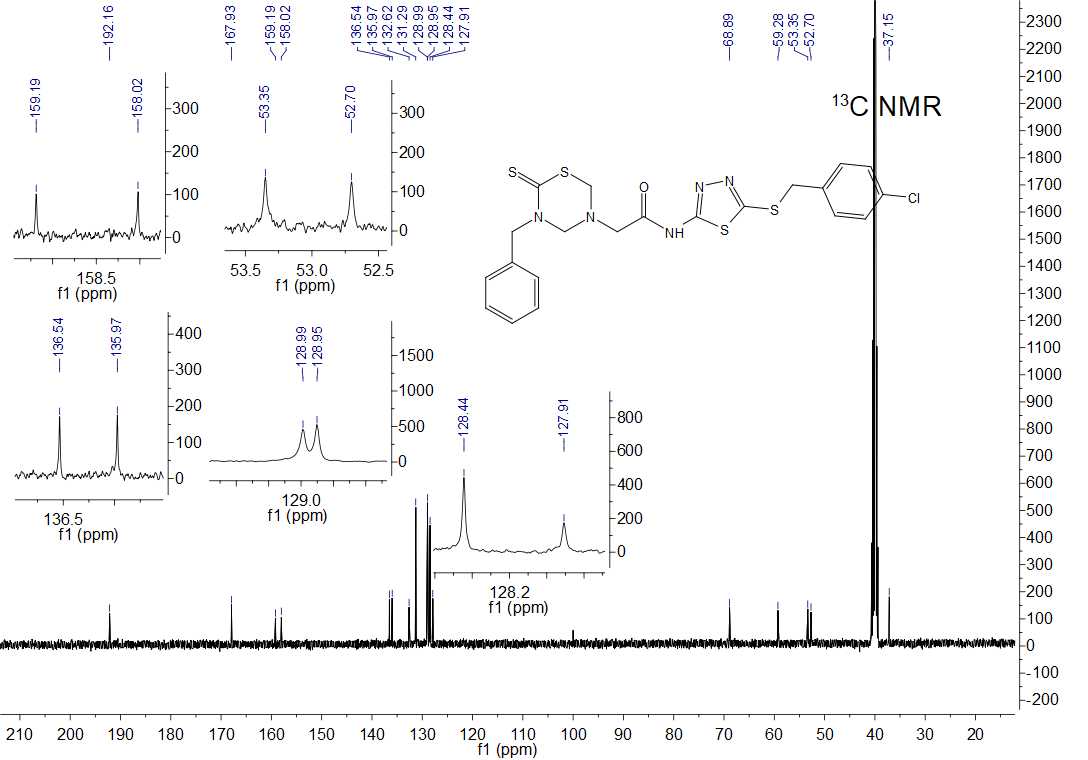


*Fig. S23* ^13^C NMR spectrum of title compound **6d**

*Fig. S24* HRMS spectrum of title compound **6d**


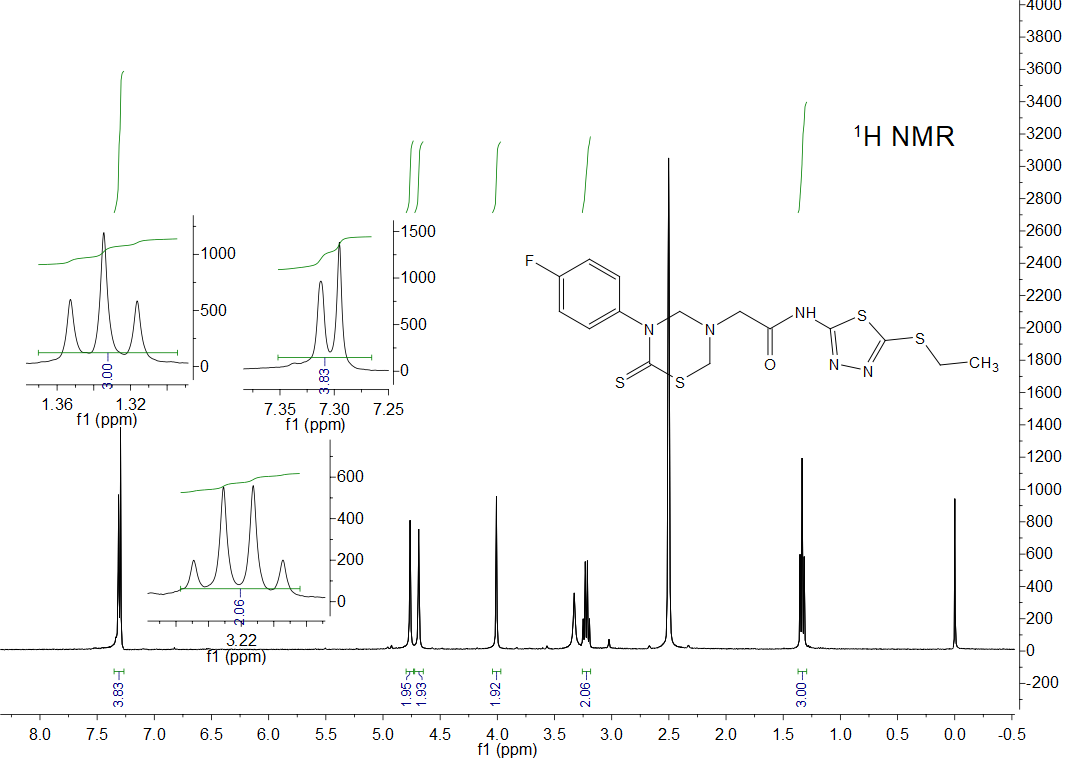


*Fig. S25* ^1^H NMR spectrum of title compound **7a**


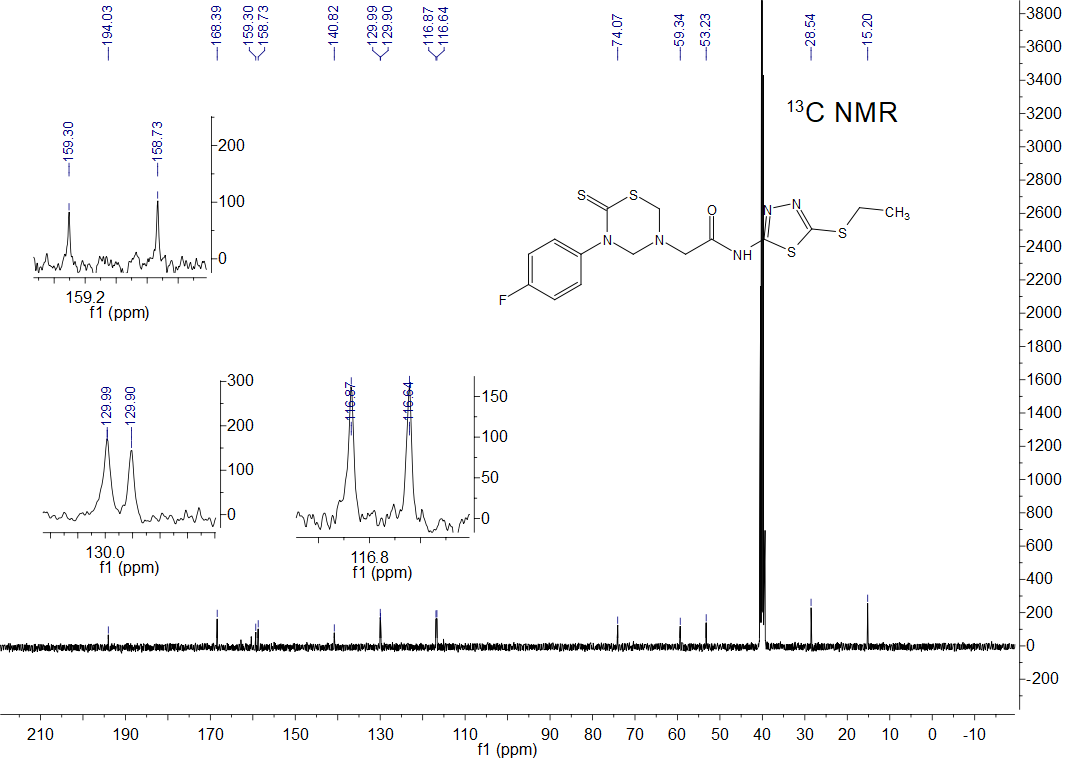


*Fig. S26* ^13^C NMR spectrum of title compound **7a**

*Fig. S27* HRMS spectrum of title compound **7a**


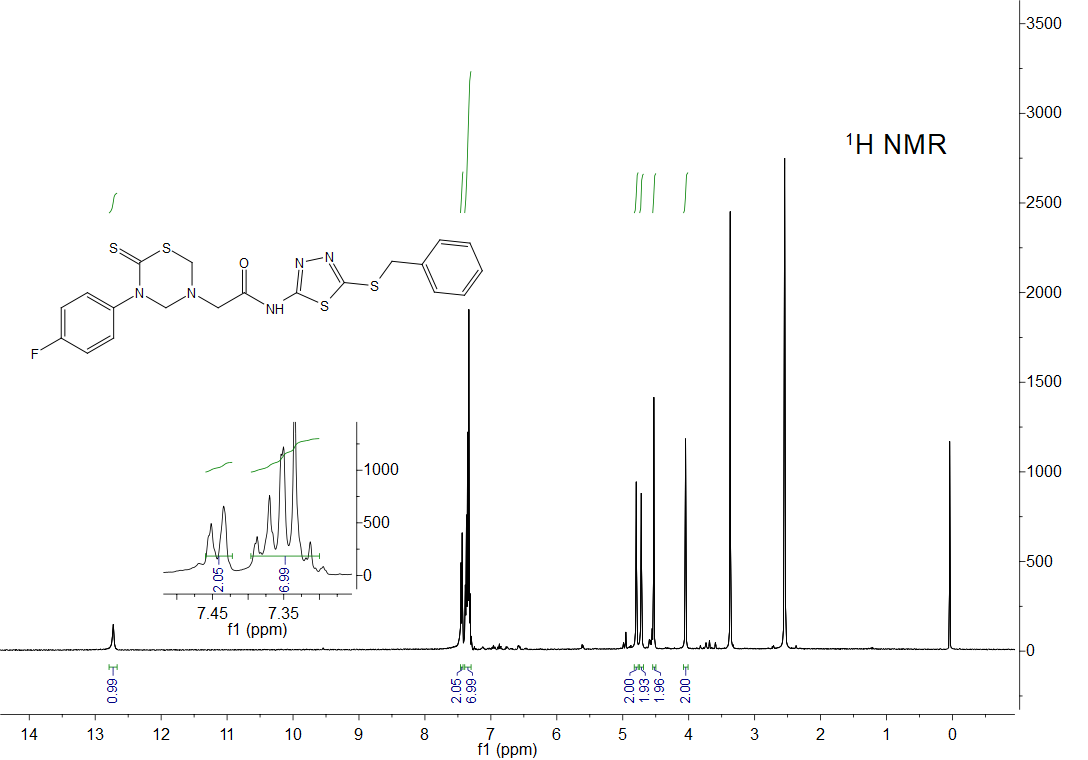


*Fig. S28* ^1^H NMR spectrum of title compound **7b**


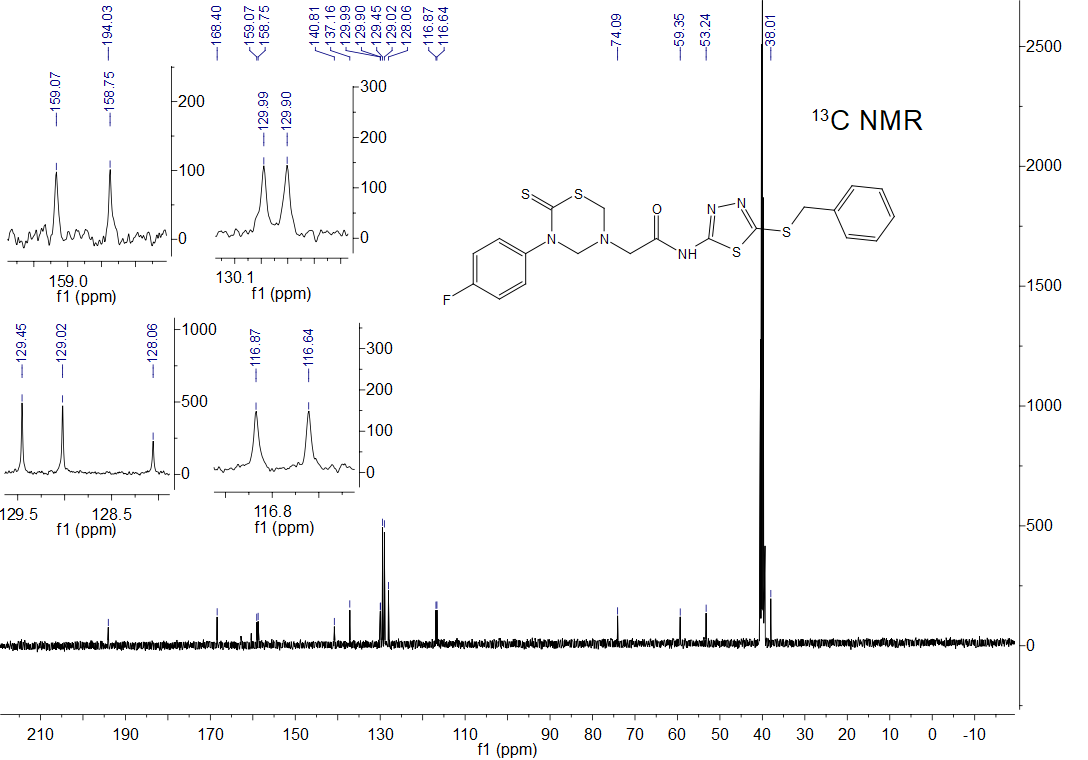


*Fig. S29* ^13^C NMR spectrum of title compound **7b**

*Fig. S30* HRMS spectrum of title compound **7b**


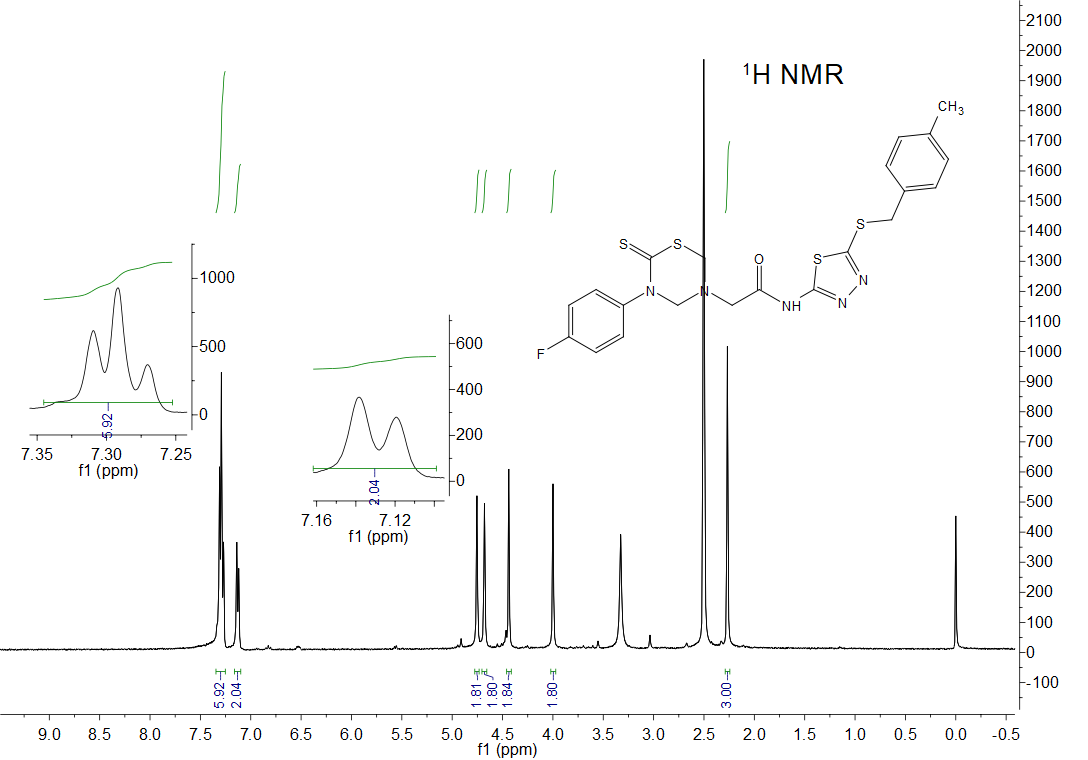


*Fig. S31* ^1^H NMR spectrum of title compound **7c**


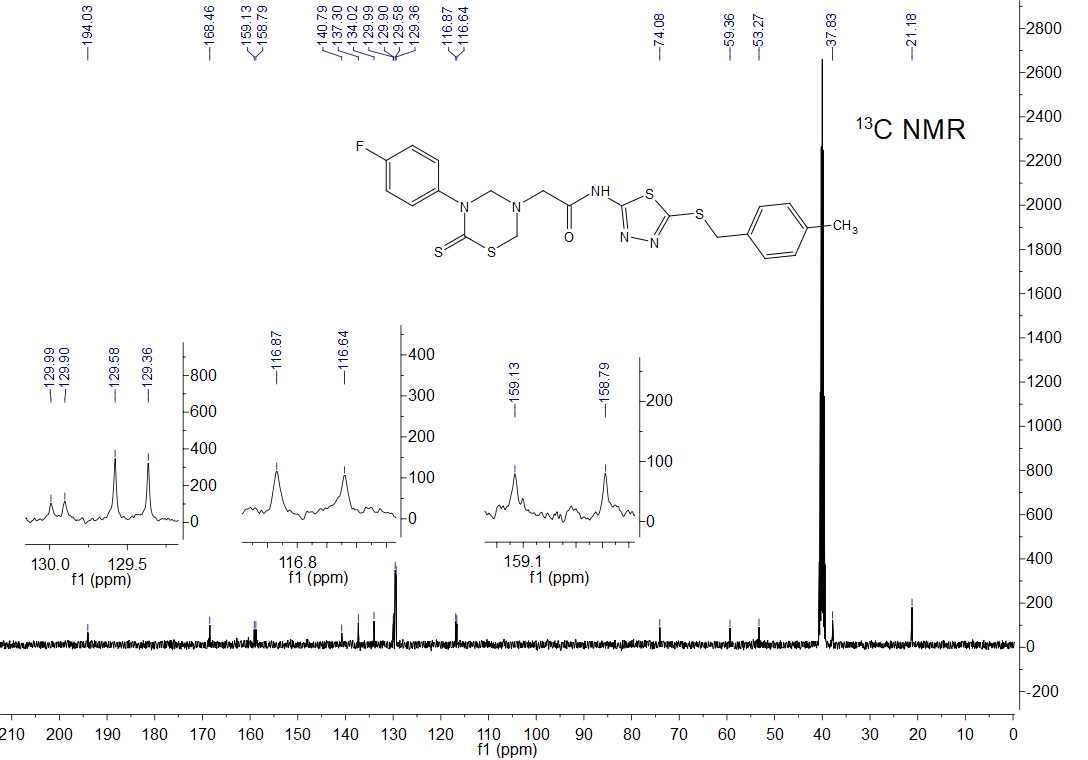


*Fig. S32* ^13^C NMR spectrum of title compound **7c**

*Fig. S33* HRMS spectrum of title compound **7c**


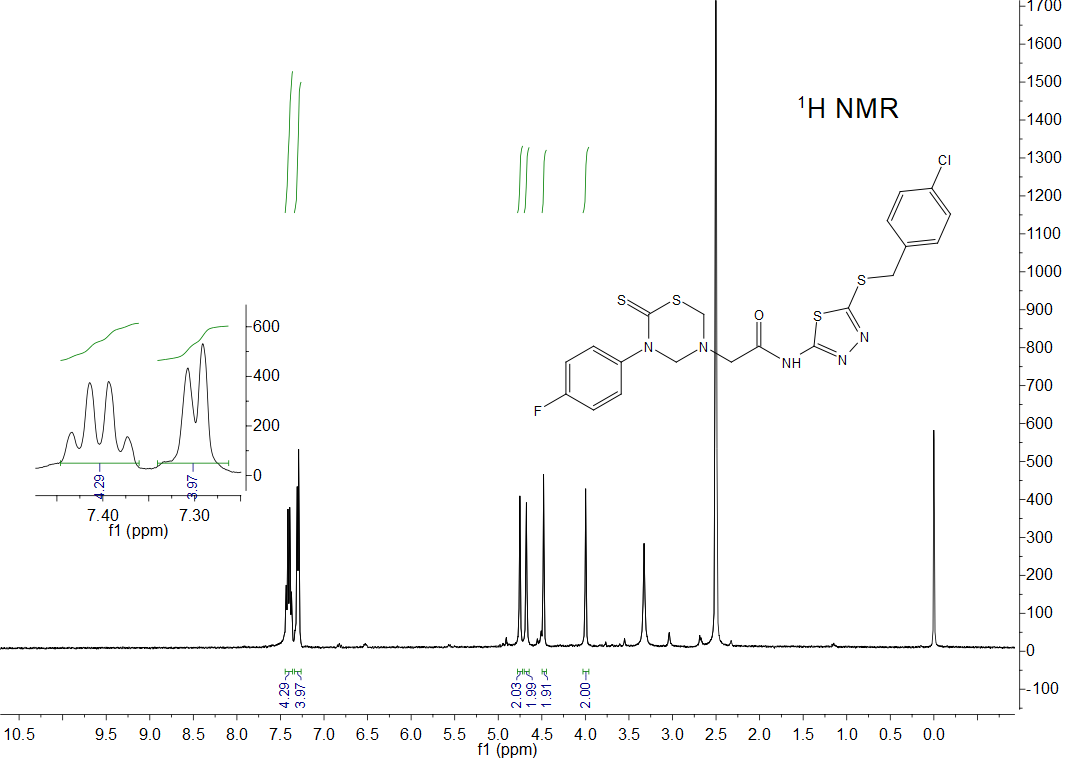


*Fig. S34* ^1^H NMR spectrum of title compound **7d**


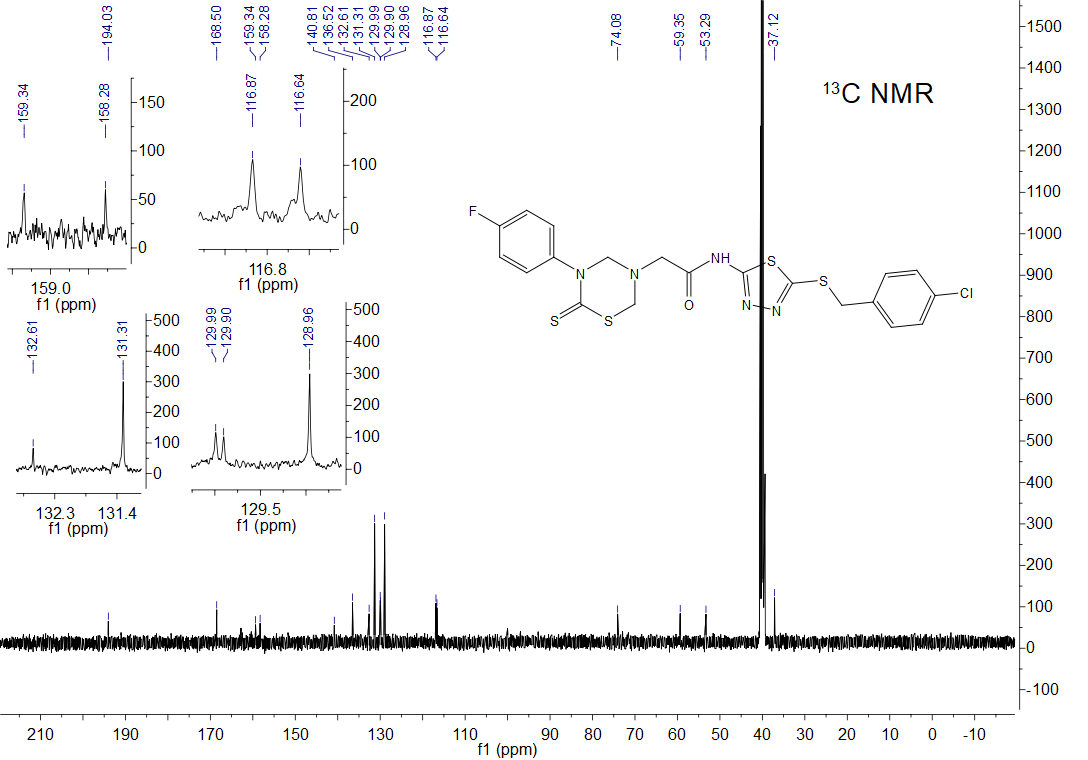


*Fig. S35* ^13^C NMR spectrum of title compound **7d**

*Fig. S36* HRMS spectrum of title compound **7d**


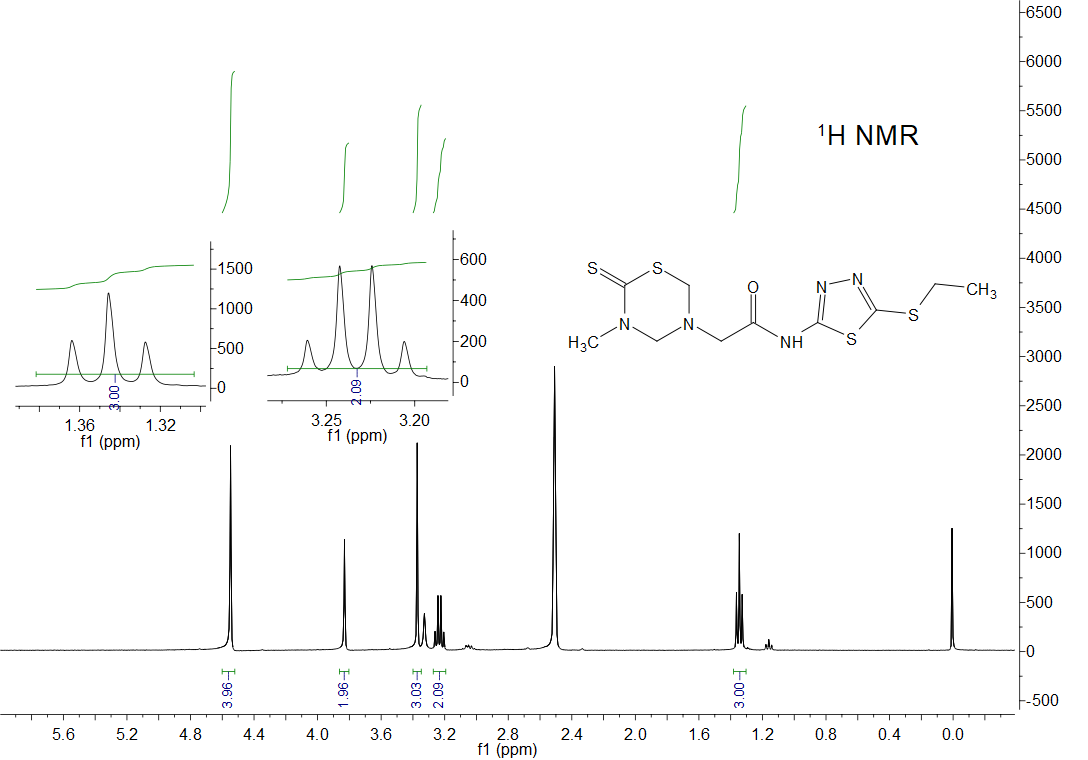


*Fig. S37* ^1^H NMR spectrum of title compound **8a**


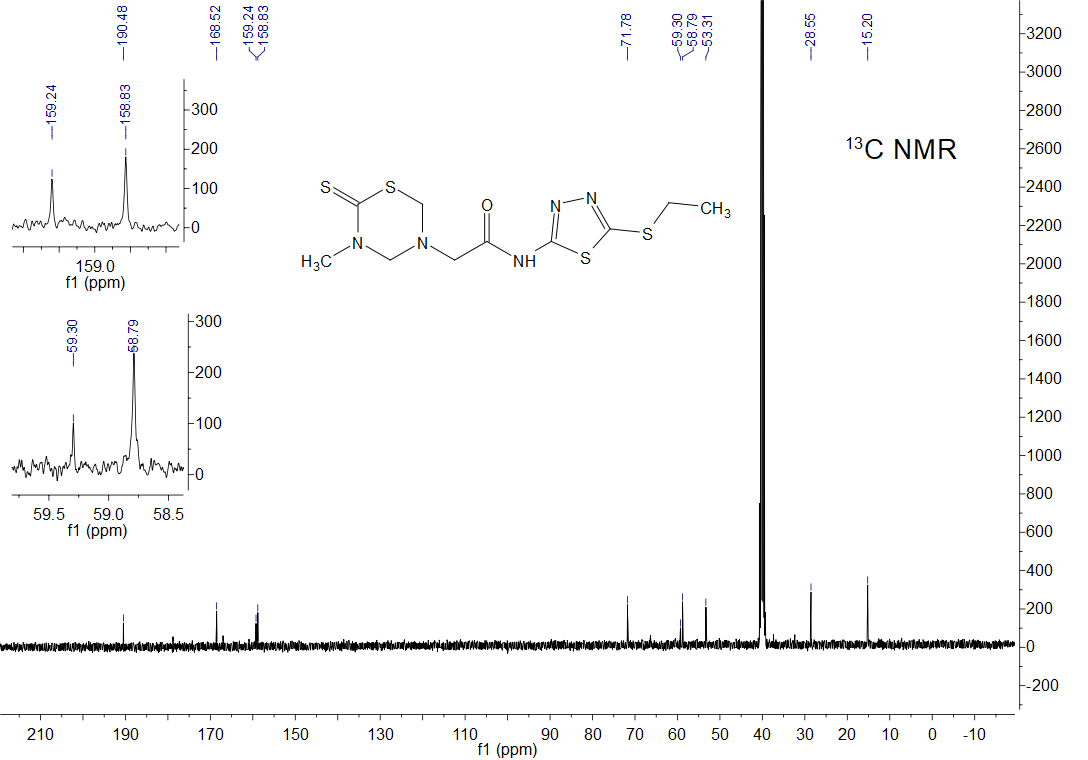


*Fig. S38* ^13^C NMR spectrum of title compound **8a**

*Fig. S39* HRMS spectrum of title compound **8a**


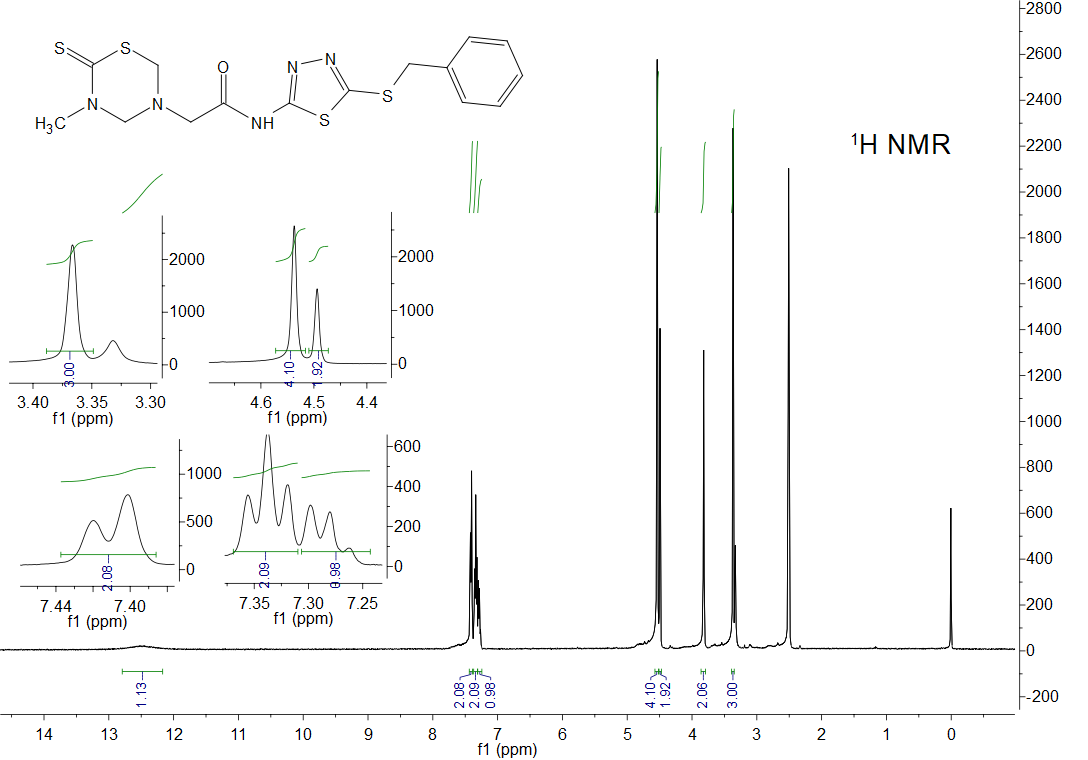


*Fig. S40* ^1^H NMR spectrum of title compound **8b**


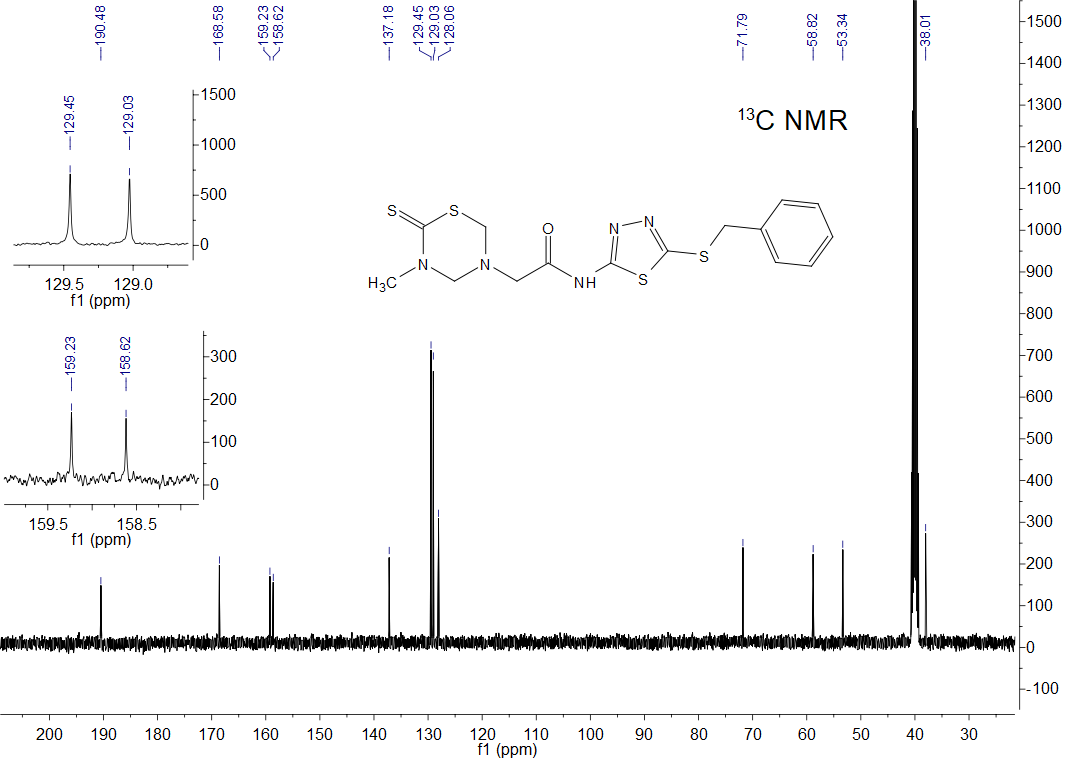


*Fig. S41* ^13^C NMR spectrum of title compound **8b**

*Fig. S42* HRMS spectrum of title compound **8b**


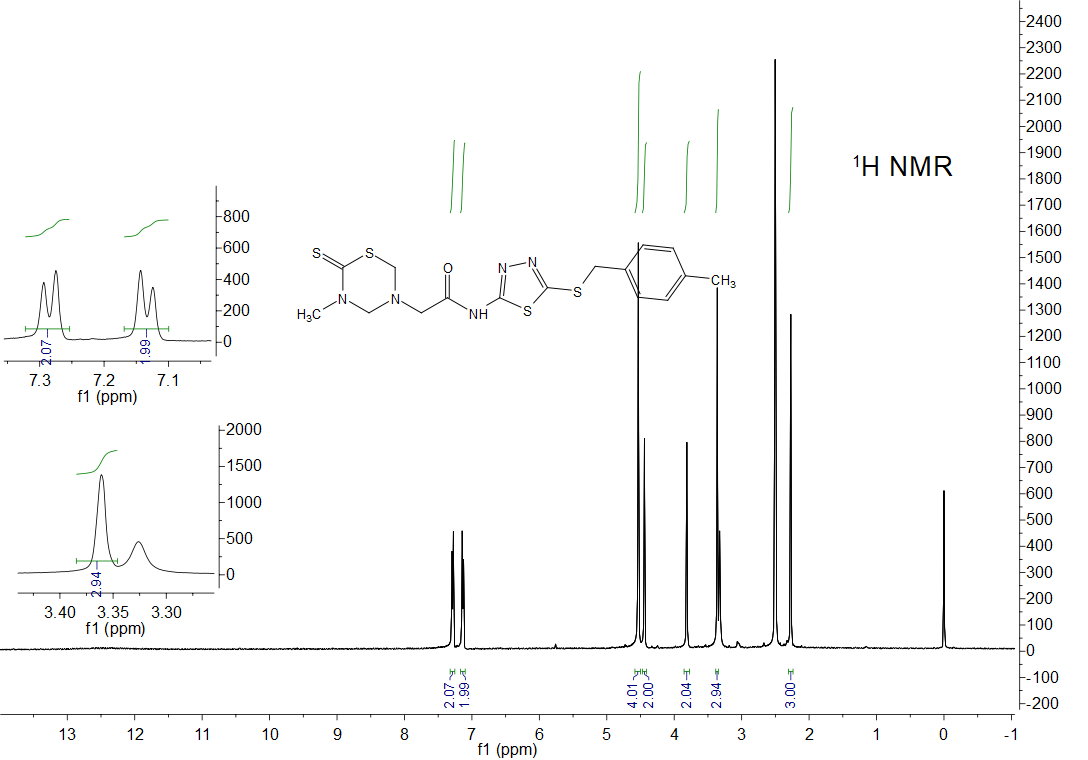


*Fig. S43* ^1^H NMR spectrum of title compound **8c**


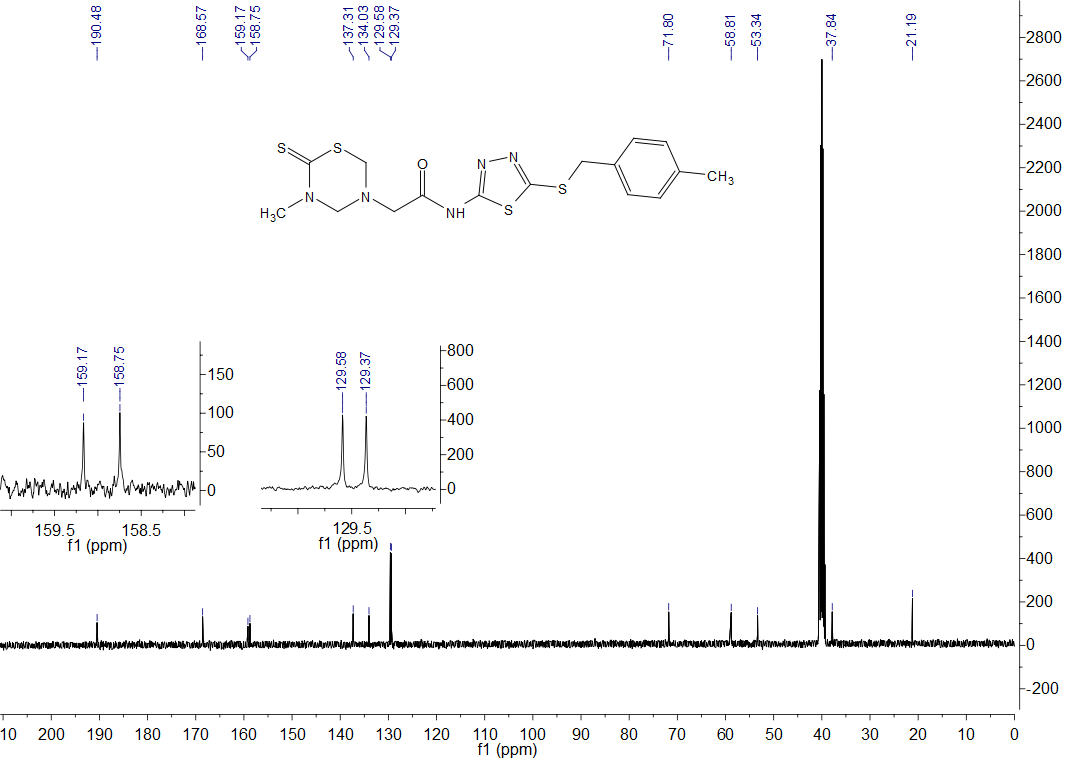


*Fig. S44* ^13^C NMR spectrum of title compound **8c**

*Fig. S45* HRMS spectrum of title compound **8c**


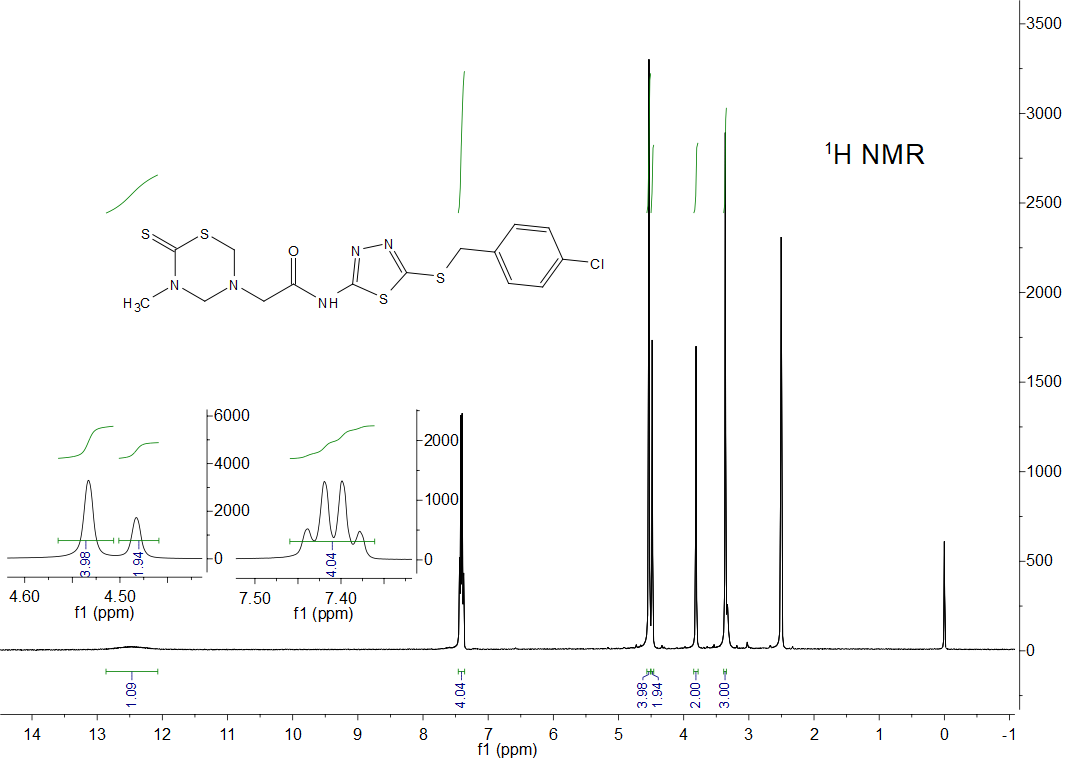


*Fig. S46* ^1^H NMR spectrum of title compound **8d**


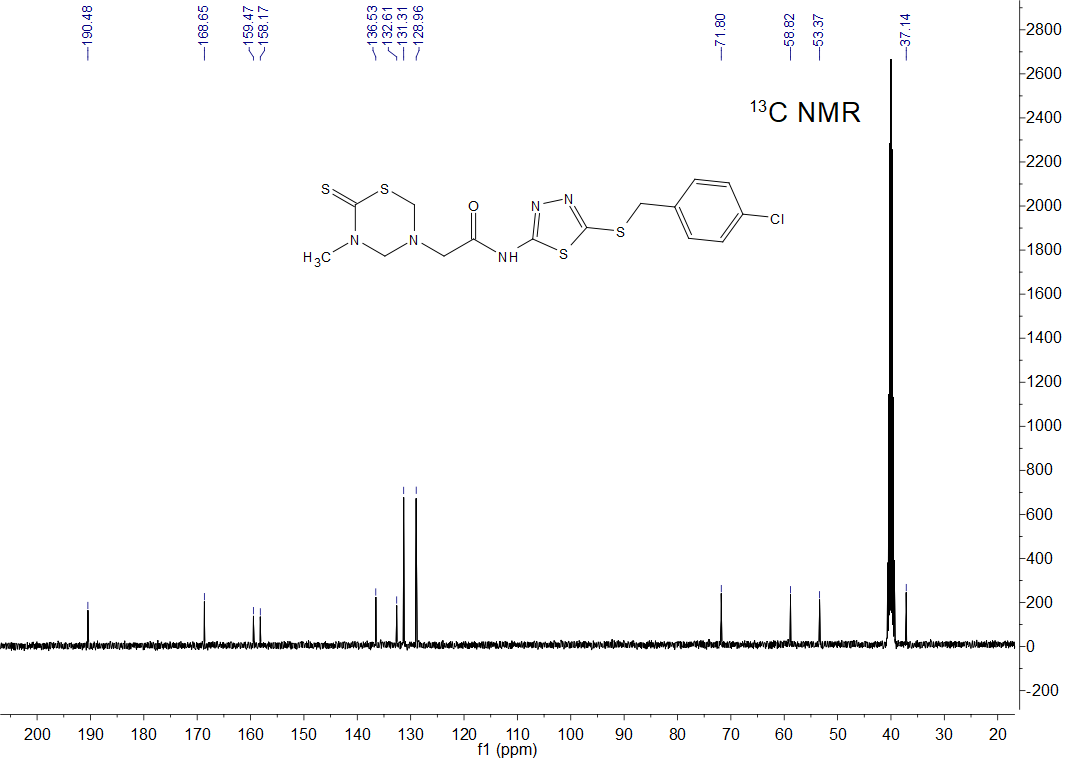


*Fig. S47* ^13^C NMR spectrum of title compound **8d**

*Fig. S48* HRMS spectrum of title compound **8d**

# 4. Crystal data of compound 8d

*Table S1* Crystal data of compound **8d**

| Empirical formula | C_15_H_16_ClN_5_OS_4_ |
| --- | --- |
| Formula weight | 446.02 |
| Temperature(K) | 100.00(10) |
| Crystal system | Orthorhombic |
| Space group | P*na*2_1_ (No. 33) |
| *a*(Å) | 9.8513(5) |
| *b*(Å) | 7.3392(4) |
| *c*(Å) | 26.2975(15) |
| *α*(°) | 90 |
| *β*(°) | 90 |
| *γ*(°) | 90 |
| *V* (Å^3^) | 1901.33(18) |
| *Z* | 4 |
| *ρ*_calc_(g/cm^3^) | 1.558 |
| *μ*(mm^‑1^) | 0.656 |
| *F*(000) | 920.0 |
| Crystal size(mm^3^) | 0.10 × 0.11 × 0.12 |
| Radiation | MoKα (*λ* = 0.71073) |
| 2*θ* range for data collection(°) | 5.8 to 56.6 |
| Index ranges | -13≤*h*≤10, -6≤*k*≤9, -33≤*l*≤31 |
| Reflections collected | 12108 |
| Independent reflections | 4013 [*R*_int_= 0.039] |
| Data/restraints/parameters | 4343/1/240 |
| Goodness-of-fit on *F*^2^ | 1.044 |
| Final *R* indexes [*I*>=2*σ* (*I*)] | *R*_1_= 0.0361, *wR*_1_= 0.0676 |
| Final *R* indexes [all data] | *R*_2_= 0.0413, *wR*_2_= 0.0706 |
| Largest diff. peak/hole ( e Å^-3^) | 0.28 and 0.31 |

# 5. The *in vitro* antimicrobial activity of the title compounds 5–8.

*Fig. S49* Anti-*Xoc* inhibition rates of the target compounds

*Fig. S50* Anti-*Xoo* inhibition rates of the target compounds

*Fig. S51* Anti-*Rs* inhibition rates of the target compounds

*Fig. S52* Anti-*Fg* inhibition rates of the target compounds

# 6. References

**Wang XB****,** **Fu XC,** **Chen M, Wang** **A, Yan JH,** **Mei** **YD, Wang MQ, Yang CL. 2019**. Novel 1,3,5-thiadiazine-2-thione derivatives containing a hydrazide moiety: Design, synthesis and bioactive evaluation against phytopathogenic fungi *in vitro* and *in vivo*. *Chinese Chemical Letters* **30**(7):1419–1422

**Wang XB, Fu XC, Yan JH, Wang A, Wang MQ, Chen M, Yang CL, Song YM. 2018**. Design and synthesis of novel 2-(6-thioxo-1,3,5-thiadiazinan-3-yl)-*N'*-phenylacethydrazide derivatives as potential fungicides. *Molecular Diversity* 1–11

**Gao YJ, Samanta S, Cui TA, Lam YL. 2013**. Synthesis and in vitro Evaluation of West Nile Virus Protease Inhibitors Based on the 1,3,4,5-Tetrasubstituted 1H-Pyrrol-2(5H)-one Scaffold. *Chem Med Chem* **8**(9):1554–1560
